# Application of parametric survival analysis to women patients with breast cancer at Jimma University Medical Center

**DOI:** 10.1186/s12885-023-11685-6

**Published:** 2023-12-12

**Authors:** Buzuneh Tasfa Marine, Dagne Tesfaye Mengistie

**Affiliations:** 1 Department of Epidemiology, Faculty of Public Health, Jimma University, Jimma, Ethiopia; 2 Department of Statistics, College of Natural and Computational Science, Jigjiga University, Jigjiga, Ethiopia

**Keywords:** Breast cancer, Stage of cancer, Estrogen receptor, Parametric survival, AFT model

## Abstract

Public health systems in both industrialized and undeveloped countries continue to struggle with the worldwide problem of breast cancer. In sub-Saharan African countries, notably Ethiopia, it is the form of cancer that strikes women the most commonly. Despite the extreme difficulties, the causes of mortality in Ethiopia have not yet been identified. In addition, little study has been done in this area. Therefore, the major objective of this analysis was to pinpoint the factors that were most responsible for the decreased life expectancy of breast cancer patients at the University of Jimma Medical Center. 552 women who had been treated for breast cancer at Jimma University Medical Center between October 2018 and December 2022 were included in this study, which used a retrospective cohort study design and five-year follow-up data. The most frequent and widely used test for comparing the probability of survival curves between several categorical independent variables was the log-rank test. Next, semi-parametric methods for multivariable analysis using the Cox proportional hazards model were used. Furthermore, a parametric strategy that includes fully parametric survival models better achieves the goal of the analysis. Among covariate, age of patient (ϕ = 254.06; 95% CI (3.95, 7.13), P-value = 0.000), patient live in urban (ϕ = 0.84; 95% CI (-0.35,-0.00), P-value = 0.047), preexisting comorbidity (ϕ = 2.46; 95% CI (0.39, 1.41), P-value = 0.001), overweight women cancer patient (ϕ = 0.05; 95% CI(-4.41,-1.57), P-value = 0.000, positive Axillary Node status cancer patient (ϕ = 0.04; 95% CI(-4.45,-1.88), P-value = 0.000), both surgery and chemotropic baseline treatment patient (ϕ = 0.53; 95% CI(-1.12,-0.16), P-value = 0.009) significantly affected the survival of women breast cancer. Age of breast cancer patient, patient education level, place of residence, marital status, pre-existing comorbidity, axillary node status, estrogen receptor, tumor size, body mass index at diagnosis, stage of cancer, and baseline treatment were found to have a significant effect on time to survive for women with breast cancer at the University of Jimma Medical Center, Oromia region, Ethiopia. However, the covariate histologic grade, number of positive lymph nodes involved, and type of hormone used were insignificant to the survival of breast cancer patients.

## Introduction

### Background of the study

Among the most common cancer in women globally, breast cancer is regarded as one of the more dangerous diseases [1]. Lobular carcinoma, or breast cancer, usually starts in one of the glands that produce milk. Breast cancer develops when cells in the breast tissue proliferate and divide out of control, resulting in a lump or tumor [2]. Breast cancer has the potential to spread to other body regions if it is not identified and treated quickly. Before dying, the majority of breast cancer patients had symptoms for more than a year [3].

In East African nations, the yearly incidence and fatality rates of age-standardized breast cancer are 33.0 and 17.9 per 100,000 women, respectively [4]. With over 2.3 million cases reported each year, breast cancer is now the second most common cancer globally in terms of death toll [5]. The death rates of breast cancer in affluent and underdeveloped nations varied significantly because of delayed manifestation and inadequate treatment [6]. In most high-income countries, over 70% of women with breast cancer were diagnosed when the disease was still in stages I and II. However, in most low- and middle-income countries, only 20 to 50% of patients receive an early diagnosis [3].

Breast cancer is the leading cause of death for women in Ethiopia. The most prevalent cause of cancer-related death among Ethiopian women is breast cancer, with a reported incidence rate of 29.6 per 100,000 women [7]. Additionally, half of breast cancer patients are young women under 40. The fact that the median age of breast cancer death is 37 years highlights the stark disparity in the number of women receiving a diagnosis [8]. Although faced with the same condition, Ethiopian women have a life expectancy that is nearly half that of American women.

Breast cancer will account for 25% of all female cancer cases in 2020, and it has been becoming more common globally, particularly in developing nations [9]. Study indicates that 15–30% of women with breast cancer may encounter distant metastases, compared to 8–10% who will experience localized recurrences [10]. In Africa, there are about 168,690 cases of breast cancer diagnosed annually, along with 74,072 deaths from the disease. Breast cancer makes up 34% of all cases and is the most common type among Ethiopian women. Every year, 60,000 additional cases of breast cancer are discovered in Ethiopia [11].

Data from the Addis Ababa cancer database study indicate that 23% of all cancer cases and 33% of cases affecting women are related to breast cancer [12].Breast cancer has significant societal ramifications because it primarily affects young or middle-aged women who are also in charge of raising their children and the family as a whole [6].

Many cross-sectional studies on the factors affecting Ethiopian women with breast cancer are currently being conducted. The study’s shorter duration made it impossible to identify the risk factors unique to patients with breast cancer [4]. Another form of survival analysis called parametric survival analysis makes use of computational frameworks to calculate the likelihood that an event will occur over time. Compared to conventional methods, this methodology has a number of advantages, such as the capacity to model intricate survival patterns and produce more precise survival result predictions [3]. The purpose of this study is to investigate the usefulness of parametric survival analysis in assessing the prognosis and survival trends of female breast cancer patients at JUMC. Therefore, the overall goal of this study was to use parametric survival analysis to identify the main factor of the lower life expectancy of breast cancer patients at Jimma University Medical Center.

The results of this study can assist medical professionals in identifying patients who are at high risk, adjusting treatment plans, and putting targeted interventions into practice in order to lower the incidence of breast cancer in this susceptible group and increase death rates. The study can also improve the management and care of women with breast cancer overall by informing public health policies and guidelines [13–16]. The results of this study may also have wider ramifications for the treatment of breast cancer in low- and middle-income nations like Ethiopia, where the disease is more common and resources are few.

## Data and methodology

### Study design and study area

A retrospective cohort study design was conducted with five-year follow-up data among women bearing breast cancer patients at Jimma University Medical Center from October 2018 to December 2022. A total of 552 women patients were included in this study. Jimma University Medical Center is one of Ethiopia’s earliest medical centers and the only teaching and referral institution in southwest Ethiopia. For the comfort of their soldiers, Italian invaders erected it in 1930 E.C. It is located 352 km southwest of the capital of Ethiopia, Jimma City. It presently works as the only imparting knowledge and referral hospital in the southwest part of the nation, treating approximately 15,000 inpatients, 160,000 outpatients, 11,000 urgent care patients, and 4500 transportation patients annually from a catchment population of about 15 million people.

#### Source of data

All female patients were diagnosed and enrolled in breast cancer treatment at the oncology units at Jimma University Medical Center from October 2018 to December 2022.

#### Target population

All women patients were diagnosed and enrolled in breast cancer treatment at the oncology unit of Jimma University Medical Center, which was the target population for this study. The individual population was of patients on treatment service from October 2018 to December 2022 at Jimma University Medical Center. All women breast cancer patients over 15 years old who fulfilled the inclusion and exclusion criteria were included in this study.

### Variables of the study

#### Response variable

Survival time was utilized as a dependent variable in this study, as was the time to death of breast cancer (if the event occurred).

#### Explanatory variable

The independent variables used in this investigation were socio-demographic, clinic-pathological, and treatment-related factors.

##### Socio-demographic factors

age at diagnosis, marital status, residence.

##### Clinic-pathological factors

BMI at the time of diagnosis, history of pre-existing comorbidity (by using the Carlson comorbidity index), axillary lymph node status, number of lymph nodes involved, estrogen/progesterone receptor status, Stage of cancer at initial diagnosis (by using the American Joint Commission on Cancer (AJCC) Manual for Staging of Cancer), histologic grade (by using the Bloom-Richardson grading system), tumor size at diagnosis, surgical margin involvement, histologic type.

##### Treatment-related factors

duration of the day from diagnosis to surgery, type of surgery, use of chemotherapy, adherence to chemotherapy, and use of hormone therapy.

Socio-demographic, clinic-pathological, and treatment-related characteristics with their variable names and categories are presented in Table 1 below.

**Table 1:** Description of covariate and code in the study

Variable	Category
**Socio-demographic factors**:	
Age at diagnosis	1 < = 40, 2 > 40
Education level Resident	1 = primary,2 = secondary ,3 = degree and above 0 = urban, 1 = rural
Marital status	1 = single, 2 = married 3 = widowed, 4 = divorced
**Clinic-pathological factors**	
Pre-existing comorbidity	1 = yes, 2 = no
BMI at diagnosis	1 = Normal, 2 = overweight
Stage of cancer	1 = I, 2 = II, 3 = III
Histologic grade	1 = grade I, 2 = grade II, 3 = grade III
Surgical margin	1 = free, 2 = involved
Axillary Node status	1 = negative ,2 = positive
Number of Positive Lymph nodes involved	0 = < 2 , 1 = >= 2
Tumor size	0 = < 2 cm, 1 = 2.5–5 cm, 2 = > 5 cm
Estrogen receptor	1 = negative, 2 = positive ,3 = not determined
Progesterone receptor	1 = positive, 2 = negative
**Treatment-related factors**	
Baseline treatment	0 = Surgery, 1 = Chemotherapy, 2 = Chemotherapy & Surgery
Duration from date of diagnosis to surgery	1 = < 30 days, 2 = >= 30 days
Adjuvant chemotherapy use	1 = yes, 2 = no
Type of chemotherapy regimen	1 = AC, 2 = Paclitaxel, 3 = Combination
Hormone therapy use	0 = yes, 1 = no
Type of hormone used	1 = Tamoxifen, 2 = Anastrozole

#### Inclusion and exclusion criteria

All female patients with breast cancer who were newly diagnosed throughout the research period and registered at Jimma University Medical Center Women who are included on medical recode records, patients with a prior diagnosis of breast cancer, and patients with a diagnosis at another referral hospital were all ineligible for the study.

#### Method of data collection

To gather information from patients’ medical records, the data intelligence format was created using relevant literature. The patient’s medical records were mined for socio-demographic, clinic-pathological, and treatment-related characteristics that were thought to play a role in breast cancer death. Data was coded, purified, entered into EPI-data 3.1, exported to STATA version 17, and then used for analysis.

#### Methods of analysis

In this study, a variety of techniques were used to analyze the survival data. The survival data in this study was analyzed using a variety of methods. For trying to describe demographic, clinical, and follow-up data in terms of central tendency, dispersion value, and frequency distribution for continuous data and categorical data, accordingly, descriptive statistics were used. The time period between the date of a woman receiving a breast cancer diagnosis and the date of death was used to compute the person-year of follow-up. It was possible to compare the survival rates of two or more groups using a nonparametric test [17]. Among these descriptive statistics were the calculation of the survival distribution from a sample and the estimation of the Kaplan-Meier survival function, which was used to calculate the survival probability of death. The log-rank test was the most typical and popular test and was used to compare the probabilities of survival curves between different categorical independent variables. The Cox proportional hazards model was then employed in semi-parametric approaches to multivariable analysis. Additionally, a parametric approach that included completely parametric survival models more effectively addresses the analysis’s objective. Independent predictors in bivariate analysis with a P-value of less than or equal to 0.25 were eligible for multivariate analysis [18]. A 95% confidence interval for the adjusted hazard ratio (HR) was used to declare a significant association between the outcome and independent variables. In multivariable analysis, variables with a P-value < 0.05 were considered statistically significant. For statistical data analysis in this research, STATA version 17 was employed.

## Result

The results presented in Table 2 show that 472 (85.5%) out of 552 illnesses reside in urban area, while 80 (14.5%) of them were raised there. 85.5% of the 472 breast cancer women living in urban areas continued to suffer from the illness (censored), whereas 85.4% had died from it (event). There were 80 women with breast cancer in rural areas, of whom 14.5% were still living (censored) and 14.6% had died as a consequence of their illness. The three main levels of education included primary, secondary, and post-secondary. The results of the study indicate that 325 patients (58.9%), 148 (26.8%), and 79 (14.3%) of the patients, respectively, completed primary, secondary, undergraduate, and postgraduate degrees. Among 325 patients with primary education levels who were diagnosed with breast cancer, 55.8% remained alive, whereas 66.5% were no longer alive. 28.7% of the patients among 148 women in secondary school continued to be alive, while 22.2% were not. In addition, out of 78 patients with a degree or higher in education, 15.5% of the breast cancer women still remain alive there (censored), whereas 11.4% of women did not survive the disease (event). Out of the 552 breast cancer patients, 277 (50.2%) were married, 153 (27.7%) were single, 112 (20.3%) were widowed, and the remaining 10 (1.8%) were divorced. From a total of 277 (50.2%) married patients, 57.6% survived, while 46.2% had events.

Of the 552 breast cancer patients, 356 (64.5%) were younger than 40 years at diagnosis, and 196 (35.5%) were older than or equal to 40 years. Of those 356 patients, 86.3% were below the age of 40 and were still surviving; the remaining 10.1% were not. At the conclusion of the experiment, 89.9% of the 196 patients in the study who had been older than 40 years had passed away from cancer of the breast, leaving 13.7% of the participants surviving.

**Table 2:** Descriptive statistics of socio-demographic factors

Variable	Category	Status				
		Survive		Death		
		Count	Percent	Count	Present	Total
Ages at diagnosis	Less than 40	340	86.3%	16	10.1%	356(64.5%)
	more than or equal 40	54	13.7%	142	89.9%	196(35.5%)
Place of residence of patient	Urban	337	85.5%	135	85.4%	472(85.5%)
	Rural	57	14.5%	23	14.6%	80(14.5%)
Education level	Primary	220	55.8%	105	66.5%	325(58.9%)
	Secondary	113	28.7%	35	22.2%	148(26.8%)
	Degree and above	61	15.5%	18	11.4%	79(14.3%)
Marital status	Single	125	31.7%	28	17.7%	153(27.7%)
	Married	186	47.2%	91	57.6%	277(50.2%)
	Widowed	76	19.3%	36	22.8%	112(20.3%)
	Divorced	7	1.8%	3	1.9%	10(1.8%)

The results of Table 3 show that 353 (63.9%) of the 552 breast cancer patients have no pre-existing comorbidity, while 199 (36.1%) have pre-existing comorbidity. Out of 353 patients with no pre-existing comorbidity, 83.8% were still alive (censored) and 14.6% were not (event). Pre-existing comorbidity affects 199 individuals, of whom 16.2% are still alive (censored) and 85.4% are not (event). Stage I, Stage III, and Stage III have been the three categories of the breast cancer stage. According to the information, the number of stage I, stage II, and stage II breast cancer patients, respectively, was 252 (45.6%), 216 (39.2%), and 84 (15.2%). Of 252 stage I cancer patients, 48.5% were still alive, whereas 38.6% had passed away (event). Among the 216 stage II people with cancer, 40.9% were still alive and 43.7% had passed away. Furthermore, out of 84 stage III cancer patients, 50.5% of them continued to be alive (censored), while 41.1% of them died from their illness (event). 301 (54.5%) of the 552 breast cancer patients had an overweight body mass index, whereas 251 (45.5%) did not. Only 36.8% of the 301 individuals with an overweight body mass index were still alive; the other 98.7% had passed away. At the conclusion of the experiment, 63.2% of the 251 patients with a normal body mass index were still alive, whereas 1.3% had gone away.

**Table 3:** Descriptive statistics of clinic-pathological factors

Variable	Category	Status				
		Survive		Death		
		Count	percent	Count	percent	Total
Pre-existing comorbidity	No	330	83.8%	23	14.6%	353(63.9%)
	Yes	64	16.2%	135	85.4%	199(36.1%)
Stage of cancer	Stage I	191	48.5%	61	38.6%	252(45.6%)
	Stage II	139	35.3%	77	48.7%	216(39.2%)
	Stage III	64	16.2%	20	12.7%	84(15.2%)
Histologic grade	Grade I	34	8.6%	24	15.2%	58(10.5%)
	Grade II	161	40.9%	69	43.7%	230(41.7%)
	Grade III	199	50.5%	65	41.1%	264(47.8%)
Surgical margin	Free	255	64.7%	117	74.1%	372(67.4%)
	Involved	139	35.3%	41	25.9%	180(32.6%)
Axillary Node status	Negative	301	76.4%	117	74.1%	418(75.7%)
	Positive	93	23.6%	41	25.9%	134(24.3%)
BMI at diagnosis	Overweight	145	36.8%	156	98.7%	301(54.5%)
	Normal	249	63.2%	2	1.3%	251(45.5%)
Number of Positive Lymph nodes involved	<=2	93	23.6%	41	25.9%	134(24.3%)
	>=2	301	76.4%	117	74.1%	418(75.7%)
Tumor size	< 2 cm	108	27.4%	43	27.2%	151(27.4%)
	2.5-5 cm	59	15.0%	36	22.8%	95(17.2%)
	>=5 cm	227	57.6%	79	50.0%	306(55.4%)
Estrogen receptor	Negative	163	41.4%	83	52.5%	246(44.6%)
	Positive	161	40.9%	54	34.2%	215(38.9%)
	Not Determine	70	17.8%	21	13.3%	91(16.5)
Progesterone receptor	Positive	255	64.7%	68	43.0%	323(58.5%)
	Negative	139	35.3%	90	57.0%	229(41.5%)

Table 4 concludes that there were three types of baseline treatment: chemotherapy, surgery, and both. In accordance with this study’s research, 193 (35%) of the patients underwent surgery, 223 (40.4%) received chemotherapy, and 136 (24.6%) received both surgery and chemotherapy as their first form of treatment. 193 women underwent surgery, and 32.5% of those treated were still alive, whereas 41.1% were not. 223 individuals were having chemotherapy; 41.6% of those undergoing treatment are still alive, compared with 37.3% of those who did not. Likewise, out of 136 patients who had received both surgery and chemotherapy, 25.9% of patients continued to be alive (censored), whereas 21.7% of breast cancer patients passed away (event).

355 (64.3%) of the 552 patients were less than 30 days from the date of diagnosis to surgery, while 197 (35.7%) were more than 30 days from the date of diagnosis to surgery. Out of 355 patients with less than 30 days from the date of diagnosis to surgery, 64.5% were still alive (censored) and 63.9% were not (event). 197 patients took more than 30 days from the date of diagnosis to surgery, of which 35.5% were still alive (censored) and 35.7% were not (event). Of the 552 breast cancer patients, 452 (82%) used hormone therapy, and 100 (18%) did not use hormone therapy. 81.2% of the 452 patients who used hormone therapy were still alive, while the remaining 83.5% were not. 18.8% of the 100 patients in the study were still living at the end of the trial, while 16.5% had passed away.

**Table 4:** Descriptive analysis of treatment-related factors to time to survive of breast cancer

Variable	Category	Status				
		Survive		Death		
		Count	percent	Count	Percent	Total
Baseline treatment	Surgery	128	32.5%	65	41.1%	193(35%)
	Chemotherapy	164	41.6%	59	37.3%	223(40.4%)
	Surgery and chemotherapy	102	25.9%	34	21.5%	136(24.6%)
Duration from date of diagnosis to surgery	< 30	254	64.5%	101	63.9%	355(64.3%)
	>=30	140	35.5%	57	36.1%	197(35.7%)
Adjuvant chemotherapy use	Yes	128	32.5%	63	39.9%	193(34.6%)
	No	266	67.5%	95	60.1%	361(65.4%)
Type of chemotherapy regimen	AC	150	38.1%	77	48.7%	227(41.1%)
	Paclitaxel	75	19.0%	26	16.5%	101(18.2%)
	Combination	169	42.9%	55	34.8%	224(40.5%)
Hormone therapy use	Yes	320	81.2%	132	83.5%	452(82%)
	No	74	18.8%	26	16.5%	100(18%)
Type of hormone used	Tamoxifen	181	45.9%	23	14.6%	204(37%)
	Anastro zole	213	54.1%	135	85.4%	348(63%)

### Non-parametric survival analysis

**Fig. 1:**
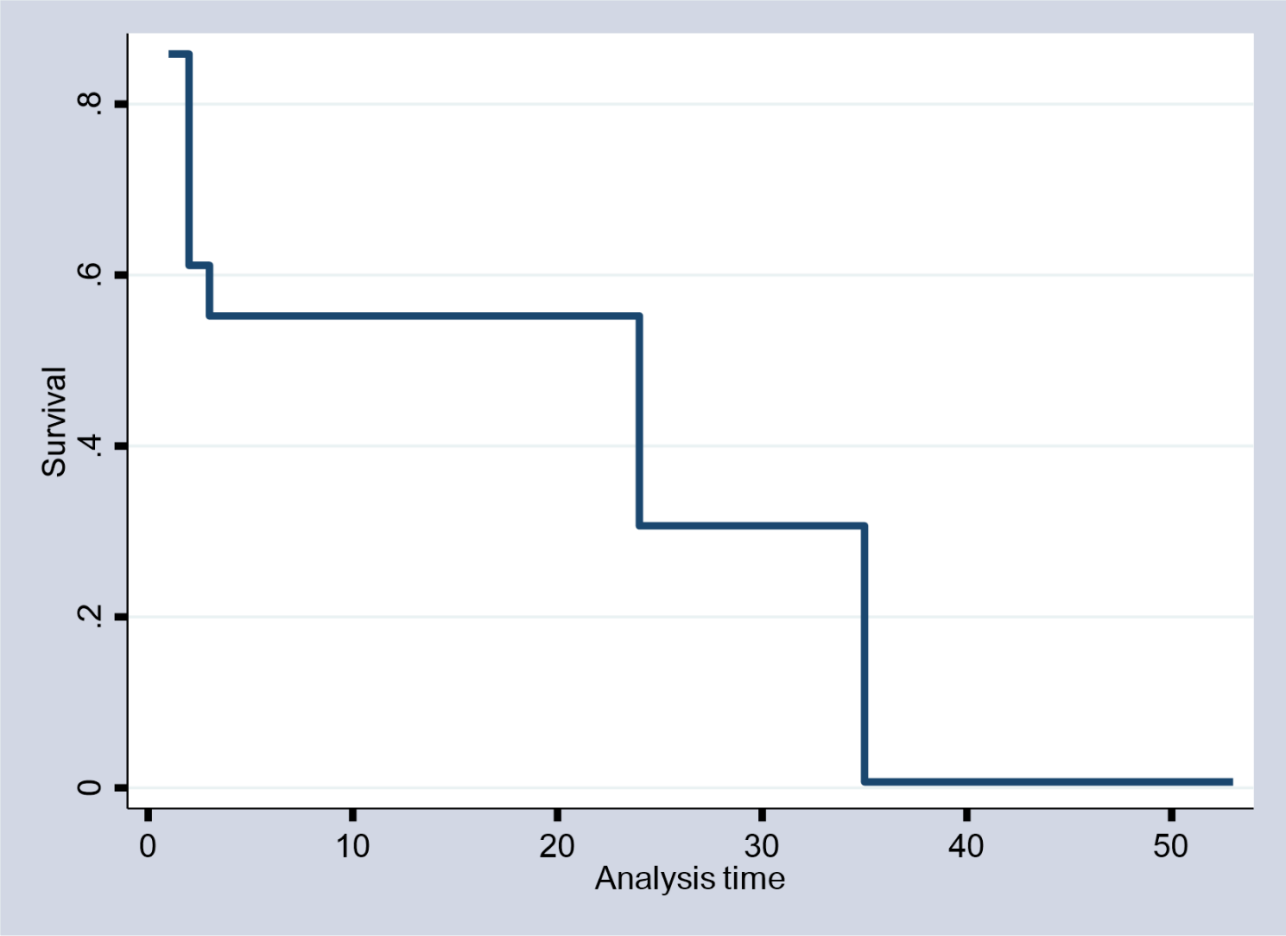
Survival plot for the time to survive breast cancer

**Fig. 2:**
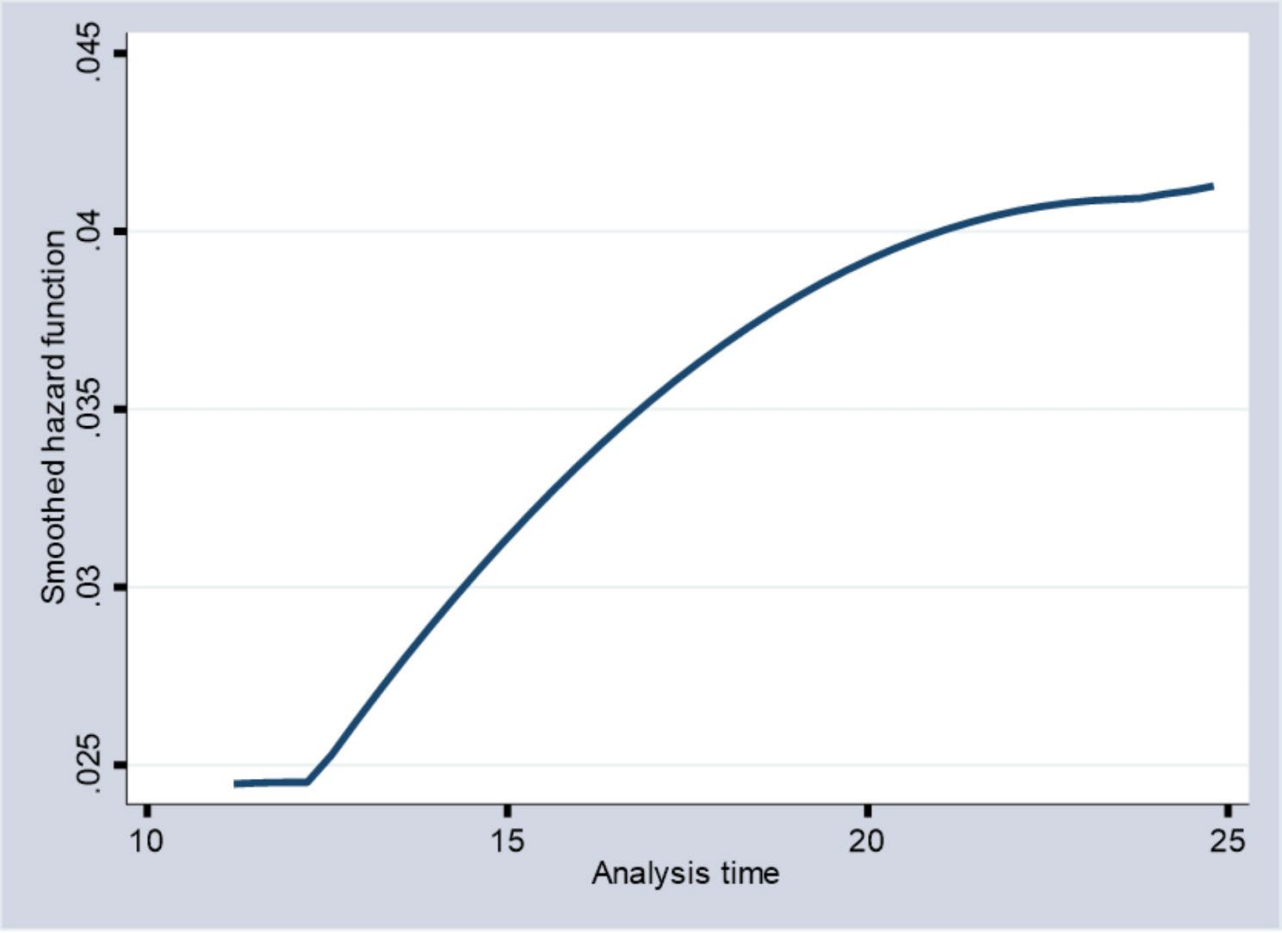
Hazard plot of time to survive of breast cancer

The Kaplan-Meir plots for the survival and hazard experience for the patient’s time to survive due to breast cancer are given in Figs. 1 and 2, respectively, for the non-parametric study. The outcome showed that the survival plot first declined at an increasing pace before occasionally declining much more. This suggests that the majority of patients with breast cancer begin therapy quite quickly. The hazard plot, on the other hand, began to climb at an increasing pace and continued to do so over time.

### Survival comparison of different groups of BM patients

**Fig. 3:**
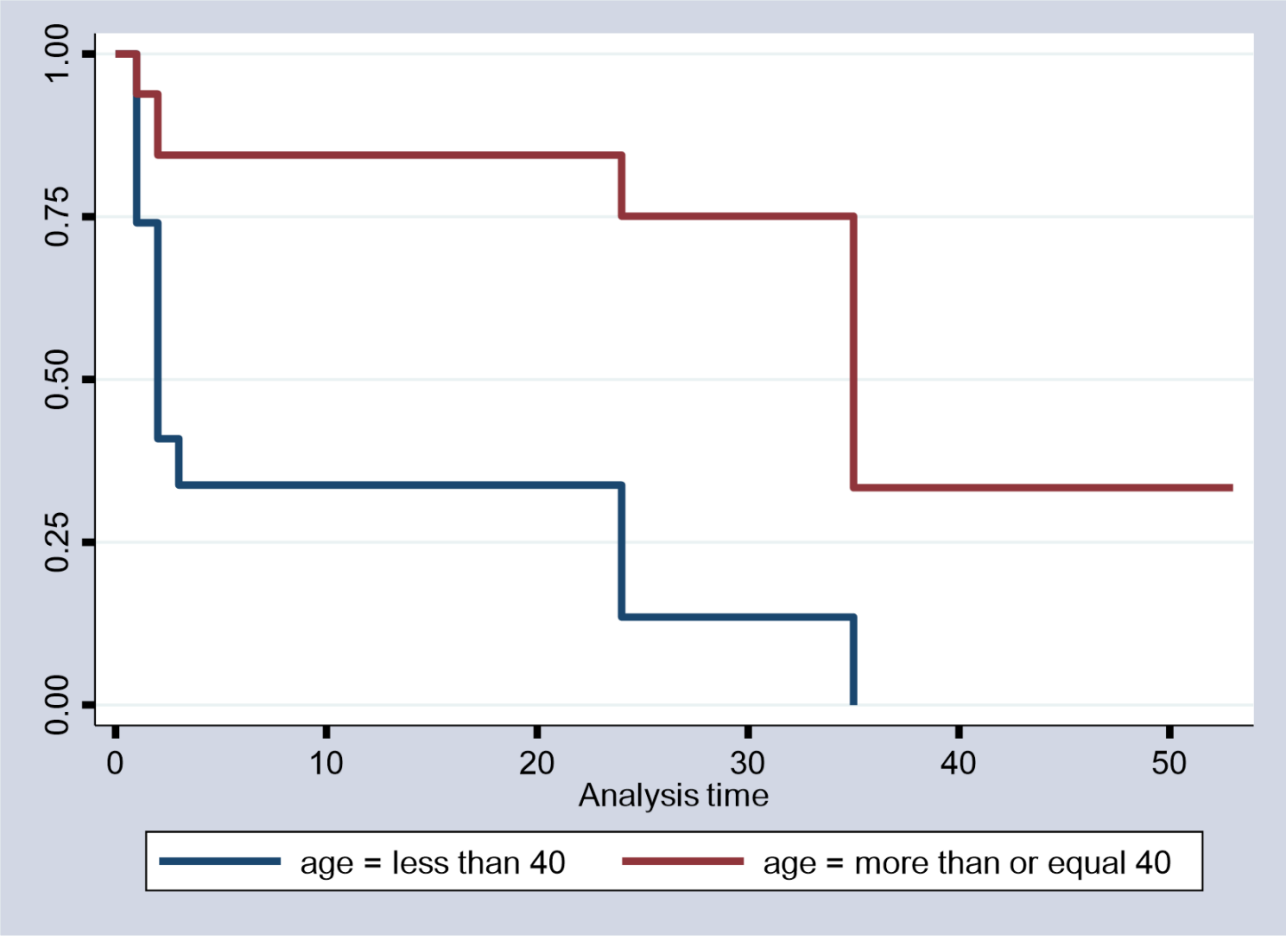
Survival time by age group of patients

As Fig. 3 indicated, the survival of the age group that is greater than 40 years is less than that of the age group that is less than 40 years, particularly at all times, whereas all are the same in the beginning and greatly different at the end, as we see in the following and this indicates that the probability of extending time to cure at a given time is greater for the patients whose age group is less than 40 years when we compare to the age group that is more than or equal to 40 years.

**Fig. 4:**
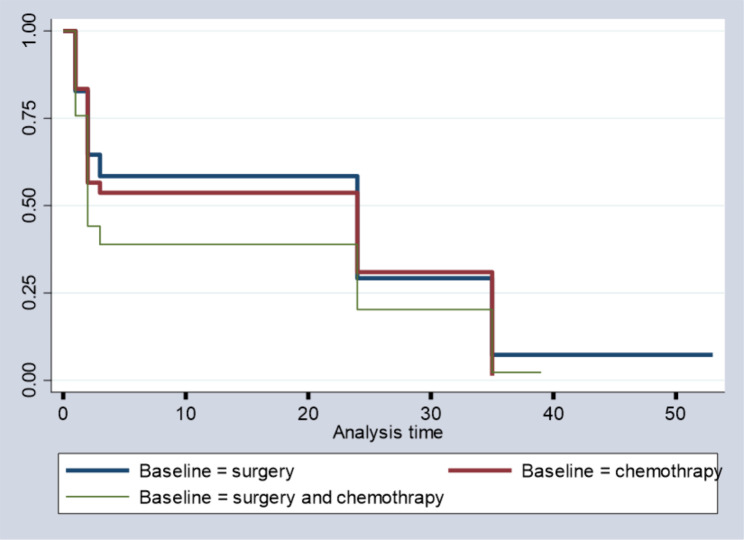
Survival time by baseline treatments

The survival of chemotherapy baseline treatment patients is higher than that of surgery and both baseline treatment patients, especially at midpoints. However, all patients’ survival rates are similar at the beginning and at the bending points, which suggests that chemotherapy baseline treatment patients have had a greater probability of getting cured at a given time than surgery and both baseline treatment users, as indicated in Fig. 4 below.

**Fig. 5:**
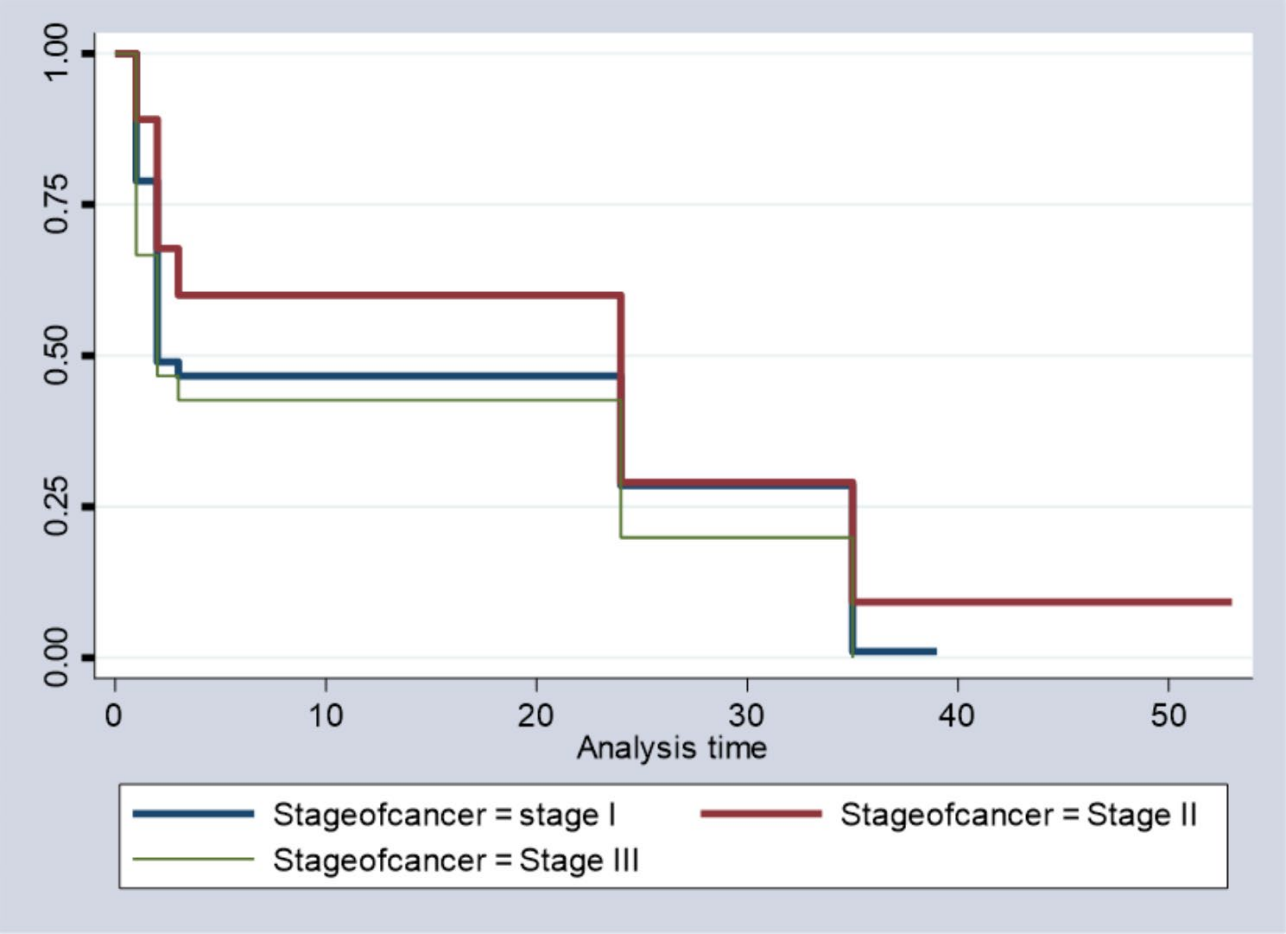
Survival time by stage of cancer

According to Fig. 5, the survival rate for the stage III cancer group of patients is lower than that of the stage I and II groups, especially in the middle times, but almost the same at the beginning and at the end, and this indicates that the probability of the curing time for stage III cancer is lower than when we compared it to the stage I and II groups, as the probability of curing time for stage I and II groups was higher than that of the stage III group.

### Multivariate AFT analysis

The factors from the model that lost significance in the multivariate analysis were eliminated using the backward elimination approach. Thus, the use of hormone treatment and the kind of chemotherapy regimen were skipped. The effect of interaction terms was also investigated and demonstrated to be statistically insignificant in the univariable Weibull AFT model at the 5% level of significance. The main impacts of the variables were preserved in the final model: age of the breast cancer patient, level of education attained by the patient, location, marital status, pre-existing conditions, estrogen receptor status of the axillary nodes, stage of cancer, baseline treatment, histologic grade, number of involved positive lymph nodes, and hormone type are all factors to consider. All AFT models are shown in Table 5, along with the associated AIC values.

Because the proportional hazard assumptions were not fulfilled for the present data analysis, the accelerated failure time (AFT), comprising the univariable and multivariable analyses, should be performed. The univariable analysis was fitted to the baseline characteristics of participants using AFT models for each covariate. For the findings on breast cancer patient survival status, AFT models of the Weibull, exponential, log-logistic, and log-normal distributions were taken into account. AIC, BIC, and log-likelihood were utilized for contrasting AFT models with Weibull, exponential, log-logistic, and lognormal distributions in order to determine which one best appropriates the data, as shown in Table 5. For this investigation, the model with the smallest AIC, BIC, and greatest log-likelihood was selected. The Weibull distribution consequently had the smallest AIC and BIC.

**Table 5:** Compares AFT models using data on breast cancer survival status using AIC, BIC, and log-likelihood criteria

Distribution	AIC	BIC	Log-likelihood
Exponential	538.426	660.823	-240.213
**Weibull**	***519.786***	**646.4037**	**-229.893**
Log logistic	526.044	652.66	-233.022
Log-normal	525.4011	652.0188	-232.701
Generalized gamma	521.659	652.4973	-229.8295
Gompertz	528.7558	655.3735	-234.378

AIC = Akaike’s information criteria, BIC = Bayesian information criterion

Table 6 shows the results of the multivariable data analysis used in this investigation. The primary effects of the covariates, which include the age of the breast cancer patient, patient education level, place of residence, marital status, pre-existing comorbidity, axillary node status, estrogen receptor, tumor size, body mass index at diagnosis, stage of cancer, baseline treatment, histologic grade, number of positive lymph nodes involved, and type of hormone used, were taken into consideration as potential predictors for the variable of interest. The factors of interest included the age of the breast cancer patient, patient education level, place of residence, marital status, pre-existing comorbidity, axillary node status, estrogen receptor, tumor size, body mass index at diagnosis, stage of cancer, and baseline treatment as statistically significant variables.

#### Age of breast cancer patient

In comparison to breast cancer patients whose age is less than 40 years, breast cancer patients who are more than 40 years old have an increasing log of survival (long survival), as indicated by the acceleration factor for age greater than 40 years being 254.06 times greater than the survival factor for that age less than 40 years (patient age less than 40 years as a reference; 254.06, 95% CI: 3.95, 7.13); P-value = 0.001).

#### Patient education level

Using the patient with primary education as a reference, it was estimated that the acceleration factor for patients with secondary education levels had been 0.43 with a confidence interval of 95% from [-1.26,-0.41], and for those who were with degrees or above, it was 0.14 with a 95% confidence interval of [-2.55,-1.43]. This indicates that a patient with breast cancer who was illiterate had a life expectancy that was greater than that of a patient who was educated.

#### Place of Residence

As shown by the acceleration factor for urban patients being 0.84 times greater than the survival factor for those living in rural areas (rural as reference; 0.84, 95% CI: (-0.35,-0.00); P-value = 0.047), patients with breast cancer who live in urban areas have a decreasing log of survival (shorter survival).

#### Marital status

The acceleration coefficient and its confidence interval of 95% of the patients who were single were 1.30 and (0.05, 0.47), respectively. This indicates that married patients survive longer than single patients, at a 5% level of significance. However, the accelerating factors and their confidence interval of 95% for a patient whose marital status had been widowed were 0.27 and (. -1.58,-1.06 with p-value 0.000), respectively. This study demonstrated that widowed patients had poorer survival times than single patients, at a 5% level of significance.

#### Pre-existing comorbidity

Patients with already existing comorbidities have an expanding log of survival (longer survival) when compared with those with breast cancer who do not. This has been shown by the acceleration factor for patients with pre-existing comorbidity being 2.64 times greater than the overall survival factor for patients with no pre-existing comorbidity (yes, as a reference; 2.64, 95% CI: -0.39, 1.41; P-value = 0.001).

#### Axillary Node Status

Patients with a positive axillary node status had smaller recovery times than patients with a negative axillary node status, or Ref (negative axillary node status), according to a significant covariate analysis involving the participants with an acceleration factor of 0.04 as well as the confidence interval for acceleration factor that exceeded one (-4.45,-1.88**).**

#### Estrogen receptor

The patients who had estrogen receptors were also significant covariates, and their confidence interval for the acceleration factor was greater than one (-0.62,-0.43) with an acceleration factor of 0.91 and a p-value of 0.000, indicating that they had less prolonged cure time than the patients who had estrogen receptors that were negative or Ref (none negative estrogen).

#### Tumour size

The acceleration factor and its 95% confidence interval for breast cancer patients with tumor size 2–5 cm were 0.40 (-1.44,-0.39), and for breast cancer patients with tumor size more than five centimeters, they were 0.39 (-1.44,-0.46); the confidence intervals for both did not include one with their respective p-values of 0.001 and 0.000. This showed that breast cancer patients with a tumor size of 2–5 cm and above 5 cm were experiencing a lower (declining) survival period than a breast cancer patient with a tumor size of less than 2 cm.

#### Body Mass Index at diagnosis

The confidence interval of the acceleration factor for overweight Body Mass Index at diagnosis patients is (-4.41,-1.57), which did not include 1, indicating that this category was also a significant determinant factor for time to cure patients from breast cancer since the acceleration factor for the overweight Body Mass Index at diagnosis patients is less than 1, which means this category had a lower curing time by a factor of 0.05 than the normal body mass index patients.

#### Stage of cancer

When using the covariate stage of cancer of a breast cancer patient in stage I as a reference, the acceleration factor of the stage of cancer of a patient in stage II was estimated to be 0.52 with a 95% confidence interval (-1.05, -0.26), and the acceleration factor of the stage of cancer patient in stage III was 0.40 with a 95% confidence interval (-1.47, -0.34). This shows that patient survival time decreased as the stage of cancer increased.

#### Baseline treatment

The confidence interval of the acceleration factor for surgery and chemotherapy patients is (-1.12,-0.16), which did not include 1, indicating that this category was also a significant determinant factor for time to cure patients from breast cancer since the acceleration factor for the surgery and chemotherapy patients is less than 1, which means this category had a lower curing time by a factor of 0.53 than the surgery-made patients.

**Table 6:** Multivariate analysis for Weibull AFT model

Variable	Coefficient	Std. err.	z	P>|z|	ϕ	[95% conf. interval]	
Age in year							
**>= 40**	5.54	0.81	6.83	0.000*	254.06	3.95	7.13
**Education**							
Secondary	-0.83	0.22	-3.86	0.000*	0.43	-1.26	-0.41
Degree and abo	-1.99	0.29	-6.97	0.000*	0.14	-2.55	-1.43
**Residence**							
Urban	-0.18	00.09	-1.99	0.047*	0.84	-0.35	-0.00
**Marital status**							
Married	0.26	0.11	2.43	0.015*	1.30	0.05	0.47
Widowed	-1.32	0.13	-9.94	0.000*	0.27	-1.58	-1.06
Divorced	0.13	0.18	0.73	0.464	1.14	-0.22	0.48
**Pre-existing comorbidity**							
Yes	0.90	0.26	3.47	0.001*	2.46	0.39	1.41
**Axillary Node status**							
Positive	-3.16	0.66	-4.83	0.000*	0.04	-4.45	-1.88
**Estrogen receptor**							
Positive	-1.01	0.20	-5.01	0.000*	0.36	-1.41	-0.62
Not determine	-0.09	0.27	-0.35	0.729	0.91	-0.62	0.43
**Tumor size**							
2–5	-0.91	0.27	-3.43	0.001*	0.40	-1.44	-0.39
> 5 cm	− 0.9496681	0.2478244	-3.83	0.000*	0.39	-1.44	-0.46
**Body Mass Index at diagnosis**							
Overweight	2.99	0.72	4.13	0.000*	0.05	-4.41	-1.57
**Progesterone receptor**							
Negative	-0.95	0.26	-3.66	0.000*	0.39	-1.46	-0.44
**Stage of cancer**							
Stage II	-0.66	0.20	-3.25	0.001*	0.52	-1.05	-0.26
Stage III	-0.90	0.29	-3.15	0.002*	0.40	-1.47	-0.34
**Histologic grade**							
Grade II	0.17	0. 0.21	0.81	0.419	1.19	-0.25 0.60	
Grade III	0.16	0.22	0.75	0.455	1.17	-0.26	0.56
**Number of Positive Lymph nodes involved**							
>=2	-0.80	0.16	-0.51	0.611	0.45	-0.40	0.23
**Baseline treatment**							
Chemotherapy	-0.20	0.21	-0.93	0.351	0.82	-0.61	0.22
Surgery and chem.	-0.64	0.25	-2.61	0.009*	0.53	-1.12	-0.16
**Type of hormone used**							
Anastrozole	-0.32	0.21	-1.53	0.125	0.73	-0.72	0.09
**Constant**	4.95	0.49	-2.97	0.000	140.55	3.96	5.89

* = Significant at 5% level, P = 1.37, λ = 0.73, θ = 0.31, chi-square = 253.95, p-value = 0.000, AIC = 519.786, BIC = 646.4037

## Discussion

The median survival duration in the current research was 15.52 (95% CI, 1.07–4.96) months. In contrast to the Gambia trial, the median time to death was 38.3 (95% CI, 32.7, 44.2) months. At 60 months of follow-up, the overall survival rate was 11.42%. This result is congruent with that of research that was done in Mali and Gambia, where the results were 13.6% and 11.9%, respectively [19]. This result, however, is less than that of previous comparable studies carried out in an Asian nation (56.04%) [20], Sweden (89%), Canada (86%), as well as the USA (88%) [21]. The survival rate for breast cancer could be lower in less wealthy countries compared to certain Western and Asian nations as a result of the lack of early screening, inadequate staging, and treatment, as well as the absence of early screening and detection [22].

The age of a patient significantly affects the relevant variable. Women with breast cancer who are older have a longer survival time from the illness. The average longevity of Ethiopian women was about 61.1 years at the time of the survey in 2010 [23]. Makes age similarly seem youthful. According to other research, breast cancer is frequently identified late and is diagnosed at high rates in young age groups in Ethiopia.

The variance in breast cancer survival in women is significantly influenced by the patient’s degree of education. Patients with breast cancer who are more educated live shorter than those who are less educated or not at all. This study runs counter to In comparison to women with primary, secondary, or tertiary education, women with an illiterate educational level had much more issues, according to an analysis of the survival time-to-death. According to several studies [24], education is positively correlated with survival time from birth to death. Others, however, discovered no correlation [25, 26]. This statistic may have developed as a result of the lack of mental healthcare and patients’ lack of financial knowledge.

According to this research, the site of residency significantly correlated with the risk of breast cancer. Women who lived in urban regions had a 0.84-fold lower risk of rural breast cancer than those who did not. This is in line with research done in the Central African Republic, which revealed that residing in cities was related to a lower risk of breast cancer [27]. The systematic review and meta-analysis, which show that living in urban regions is linked to a greater risk of breast cancer, contradict this finding [28]. This might be a result of disparities in the population’s economic standing, levels of education, and knowledge of risk factors in urban area.

The patient’s **pre-existing comorbidity** has an important effect on the long-term outlook of breast cancer. Patients who already had comorbid conditions had a greater survival rate than those who did not. This outcome was in line with research done in Ethiopia [28]. This may be because individuals with co-morbidities are physiologically more susceptible to the deleterious effects of current or prior malignancies’ treatments [29]. Furthermore, co-morbidities are linked to variations in the morphology, histology, differentiation, and proliferative state of cancer cells [30]. Additionally, individuals had a greater probability of dying if there were more lymph nodes involved that were positive. This occurs as a result of a rise in relapse rates and a fall in survival rates [5].

Breast cancer survival was significantly correlated with the baseline therapy. Those receiving both surgery and chemotherapy as baseline treatments had poorer survival times than those receiving only surgery. This study, which was backed by Olivotto’s study, revealed that chemotherapy results in a 10% increase in overall survival rates for women under the age of 50 and at least a 4% increase in overall survival rates for women over the age of 50 [2]. Our analysis’s findings revealed that chemotherapy was linked to a higher risk for patients with stage I cancer who were older than 50. Chemotherapy improves stage IV cancer patients’ overall survival rates, according to Anna’s study from 2009 [11]. For women who have early-stage breast cancer, surgery is an especially successful kind of therapy. Surgery’s long history of being used frequently to treat cancer may be the reason for the greater risk reduction attributed to it compared to other therapies. Comparing surgical removal of the tumor to other forms of treatment, it is easier and more efficient. Surgery can be helpful in the later stages of cancer for local tumor administration and tumor reduction in size [31].

Cancer of the breast is greatly affected by the patient’s cancer stage. Patients in stages II and III had more severe survival rates compared with those in stage I. Similar findings revealed that individuals with stage I and stage II cancer who underwent surgery fared better than those who did not. Furthermore, radiation and chemotherapy patients with stage I and stage II cancer had poorer survival rates than those who did not get these therapies [32]. Chemotherapy or other therapies have the potential to be used on patients with stage III breast cancer to decrease the tumor’s size and make surgery easier. After surgery, hormone treatment or a combination of both may be used to lower the chance of a recurrence of cancer. As a result, based on the individual circumstances of each patient with stage III breast cancer, numerous treatment choices, including each of the four types of therapies or combinations of them, may be taken into consideration [33].

The length of breast cancer survival is significantly influenced by the patient’s BMI at the time of diagnosis. Those who have an overweight index of body mass have a more serious probability of surviving compared to those with a normal body mass index. In a similar vein, this study discovered that having a BMI over 25 kg/m2 or being overweight was associated with a higher risk of breast cancer than having a low BMI. This outcome was in line with the earlier systematic review and meta-analysis, which determined that a body mass index of 5 kg/m^2^ was linked to a 2% increase in the risk of breast cancer. Additionally, sedentary lifestyles are linked to higher cancer risk factors in those with greater body mass indices [31].

## Conclusion

The goal of this study was to analyze factors related to the time to survive breast cancer among women patients at Jimma Medical Center using various parametric AFT models. The dataset used for this study was a dataset of breast cancer with time to survive for patients (duration of discharged patients from hospital), which we obtained from the Jimma Medical Center in Oromia Regional State of Ethiopia. To determine factors associated with the survival of patients with breast cancer, various parametric AFT models were applied.

Among these using AIC, the most appropriate statistical model, which describes the time to survive of patients with breast cancer, was the Weibull AFT model. The result of the Weibull AFT models revealed that age of the breast cancer patient, patient education level, place of residence, marital status, pre-existing comorbidity, axillary node status, estrogen receptor, tumor size, body mass index at diagnosis, stage of cancer, baseline treatment, histologic grade, number of positive lymph nodes involved, and type of hormone used were taken into consideration as potential predictors for the variable of interest. Age, Patient Education Level, Location, Marital Status, Pre-Existing Comorbidity, Axillary Node Status, Estrogen Receptor, Tumor Size, Body Mass Index at Diagnosis, Stage of Cancer, and others were the parameters of interest. In Ethiopia’s Jimma Medical Center’s Oromia area, it was discovered that initial therapy had a substantial impact on the amount of time that women with breast cancer survived. The covariates of histologic grade, the number of lymph nodes involved that were positive, and the kind of hormone utilized had no bearing on the survival of breast cancer patients.

The age of the patient in the year, pre-existing comorbidity, and marital status exhibited an encouraging association with cancer breast patients’ survival among the most significant predictor factors. On the other hand, patient education level, place of residence, axillary node status, estrogen receptor, tumor size, body mass index at diagnosis, stage of cancer, baseline treatment, histologic grade, number of positive lymph nodes involved, and type of hormone used had a negative association with the survival of breast cancer. The estimated median survival of female breast cancer patients was found to be 15.52 months.

### Strengths and limitations of the study

This study has implications for breast cancer patients, cancer incidence, doctors, and policymakers in terms of identifying breast cancer determinants. When creating strategies to lower the incidence of breast cancer in women, public health must include the risk factors that were identified in this study. Furthermore, via health education and behavior change programs, the risk factors indicated in this study, such as physical activity and breastfeeding, can be altered. Environmental pesticide exposure was excluded from the study due to the difficulty in forecasting it and the high cost involved in accomplishing so. In addition, variables like women smoking, women drinking alcohol, and triple-negative breast cancer were not included in the study because they were not recoded and available in the patient card.

## Data Availability

The corresponding author can provide access to the datasets used and analyzed during the current investigation upon reasonable request.

## Abbreviations

AFT: Accelerate frailty time
AIC: Akaike Information Criteria
BIC: Bayesian information criteria
BMI: body mass index
CI: confidence interval
AJCC: American Joint Commission on Cancer
JUMC: Jimma University Medical Center
